# Laparoscopic Transgastric Resection of a Large Gastric GIST: A Case Report and Review of Literature

**DOI:** 10.1055/s-0041-1739116

**Published:** 2021-12-15

**Authors:** Eham Arora, Jaini Gala, Aditya Nanavati, Arun Patil, Ajay Bhandarwar

**Affiliations:** 1 Department of General Surgery, Grant Medical College & Sir JJ Group of Hospitals, Mumbai, Maharashtra, India; 2 Department of Hepatobiliary and Transplant Surgery, Jupiter Hospital, Thane, Maharashtra, India

**Keywords:** gist, gastric tumor, laparoscopic resection, trans-gastric surgery

## Abstract

**Introduction**  Gastrointestinal stromal tumors (GIST) are the most common mesenchymal tumors of the gastrointestinal (GI) tract. Their primary treatment is surgical.

**Case Report**  Here we report a case of a 36-year-old male patient who was being evaluated for weakness, anemia, and melena. Upper GI endoscopy showed a mass projecting into the lumen and an abdominal computed tomography (CT) confirmed a well-defined mass close to the lesser curvature on the posterior wall. An endoscopic ultrasound-guided fine needle aspiration suggested a diagnosis of GIST. After optimization, the patient was taken up for a laparoscopic transgastric resection of the GIST. The resected specimen measured 9.5 × 8.5 × 7.5 cm. Postoperatively, the patient recovered well and was discharged by the fifth postoperative day.

**Discussion**  While traditionally, open surgery has been advocated for GISTs, for fear of spillage and peritoneal seeding, the role of minimal access surgery has been growing in recent years. The use of a transgastric approach avoids the potential complication of luminal stenosis following a wedge resection of a tumor close to the cardia. Because lymphadenectomies are rarely required and local invasion is uncommon, a wide local resection is usually curative. Thus, a laparoscopic approach can be considered as the first line in uncomplicated GISTs, irrespective of tumor size.

 Gastrointestinal stromal tumors (GISTs) are the most common mesenchymal tumors of the alimentary tract. They arise from the interstitial cells of Cajal, which are the pacemaker cells of the gastrointestinal (GI) tract. While they can arise anywhere within the GI tract, the most common site of involvement is the stomach (56%), followed by the small intestine (32%) and then the colon and rectum (6%). 1 These tumors may range from small, benign lesions to large, necrotic and hemorrhagic masses with metastases. They are frequently asymptomatic and may be detected incidentally on imaging studies obtained for other indications. Patients with symptomatic GISTs often suffer from vague abdominal discomfort, early satiety, nausea, and upper GI bleeding, producing melena and anemia. Suspected GISTs are usually evaluated with computed tomography (CT) and magnetic resonance imaging (MRI) scans, where they appear as heterogenous, hypervascular, and enhancing masses, which displace rather than invade adjacent organs. On upper GI endoscopy, they appear as smooth submucosal elevations. Unlike other malignant lesions in the GI tract like adenocarcinomas, the prognosis after a malignant GIST is favorable. The mainstay of treatment is complete surgical resection. Traditionally, this was accomplished by open surgery, but in recent years, the role of minimally invasive methods is growing. Here, we report a case with a large GIST arising from the posterior gastric wall, which was resected via a laparoscopic transgastric approach. 

## Case Report

**Fig. 1:**
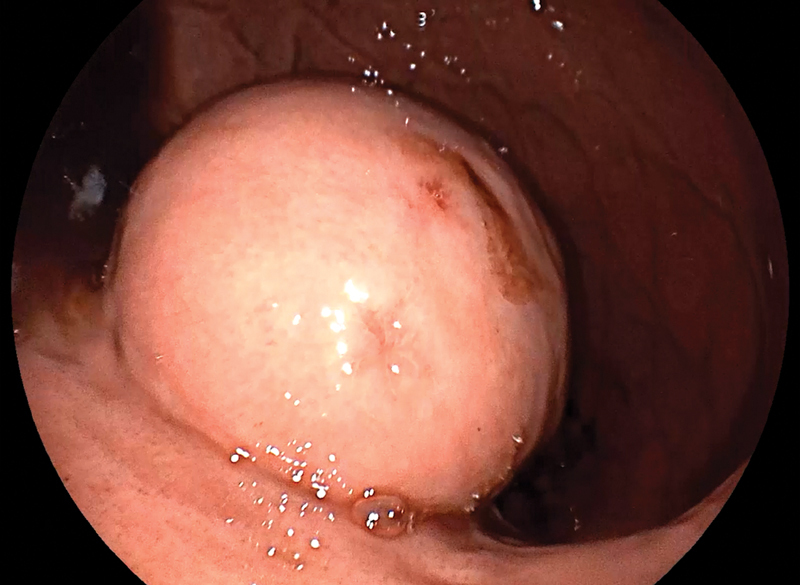
Endoscopic picture showing a large mass, arising from the posterior gastric wall, projecting into the gastric lumen.

**Fig. 2:**
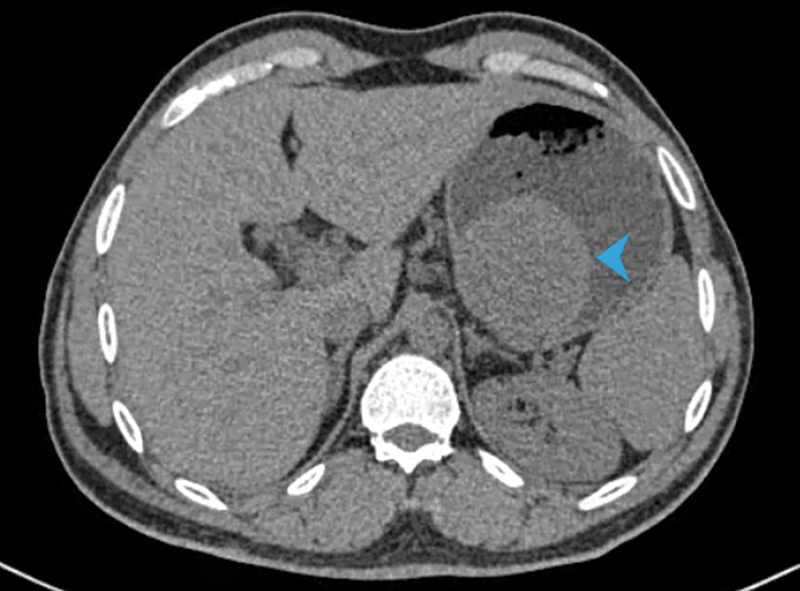
Abdominal computed tomography (CT) scan. Blue arrowhead points to the well-circumscribed mass arising from the posterior gastric wall.

 A 36-year-old male patient presented with anorexia, easy fatigability, and melena for 3 months. His clinical examination was largely unremarkable. We found melena on a digital rectal examination. An upper GI endoscopy revealed a large mass projecting into the gastric lumen ( Fig. 1 ), arising from the posterior gastric wall. The lesion was closer to the lesser gastric curvature than the greater curvature. At the summit, the mass suffered from a bleeding mucosal ulcer, the cause of his melena. Several endoscopic biopsies were inconclusive, consisting of only gastric mucosal tissue within the biopsy specimens. An endoscopic ultrasound-guided needle aspiration revealed stromal cells on cytology. An abdominal CT demonstrated a well-defined, intramural mass arising close to the lesser gastric curvature with a small mucosal defect at the superolateral aspect of the lesion ( Fig. 2 ). The soft-tissue fat planes were intact and there were no enlarged or abnormally enhancing lymph nodes. The patient's severe anemia was corrected with several preoperative transfusions. 

**Fig. 3:**
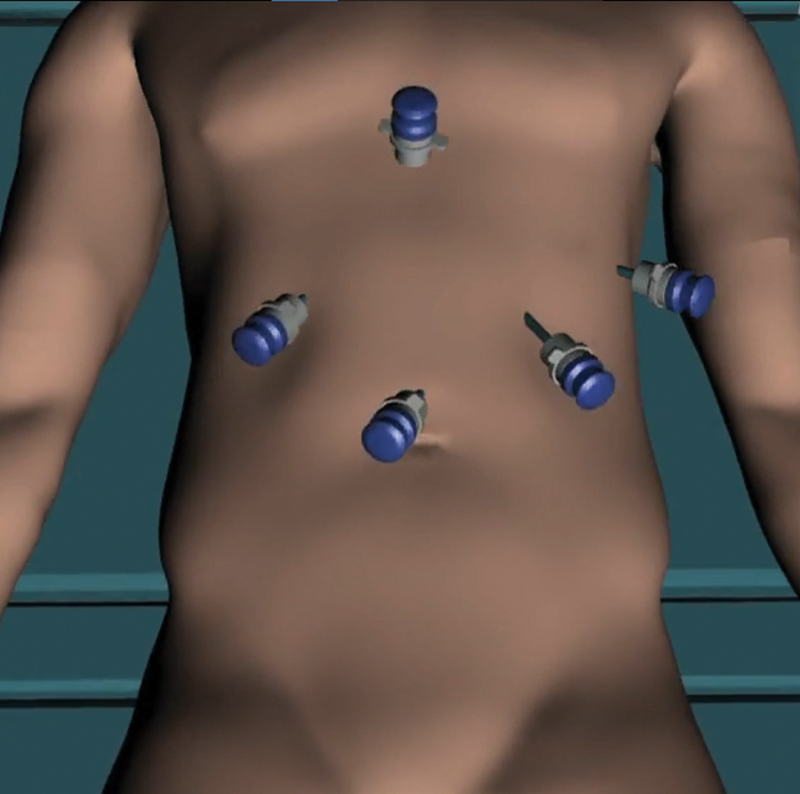
The laparoscopic port positions utilized at surgery.

**Fig. 4:**
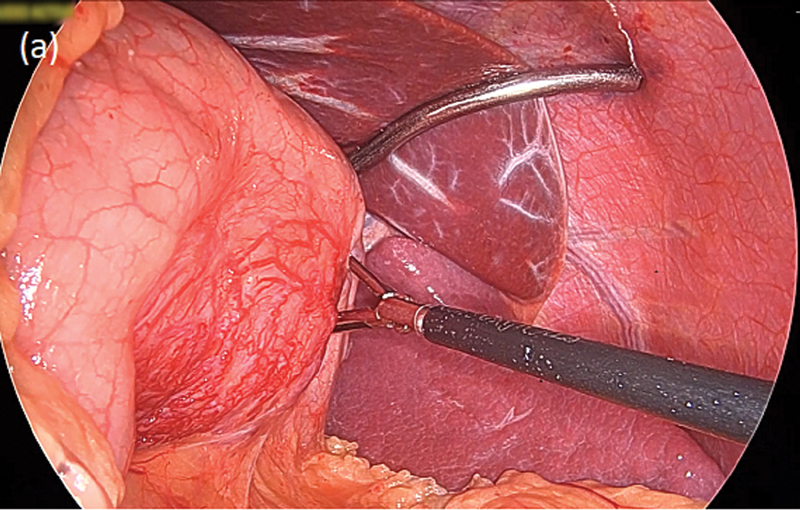
Intraoperative picture showing the dissection of the posterior attachments of the stomach. The tumor base, seen next to the grasper, has remarkably increased vascular markings as compared with adjacent gastric tissue.

**Fig. 5:**
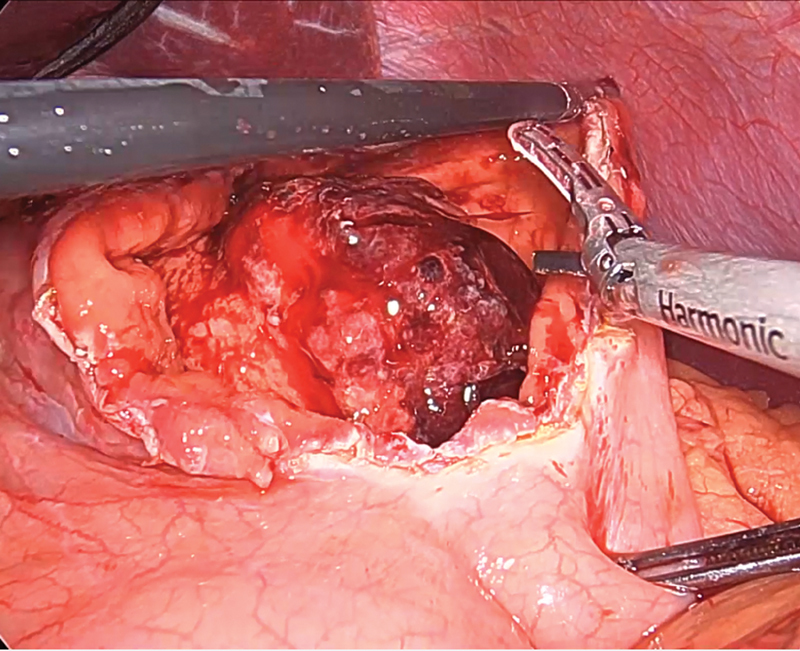
The gastric tumor coming into view through the anterior gastrotomy.

**Fig. 6:**
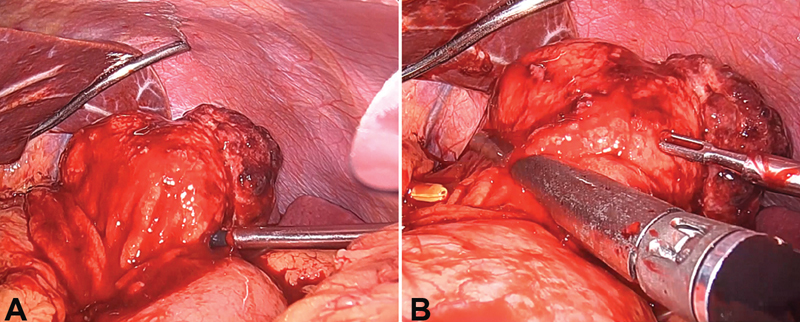
( **A** ) Tumor is pivoted onto an atraumatic bowel grasper, allowing the stomach wall at its base to be tented up; ( **B** ) an endostapler being fired across the stomach wall close to the base of the tumor.

**Fig. 7:**
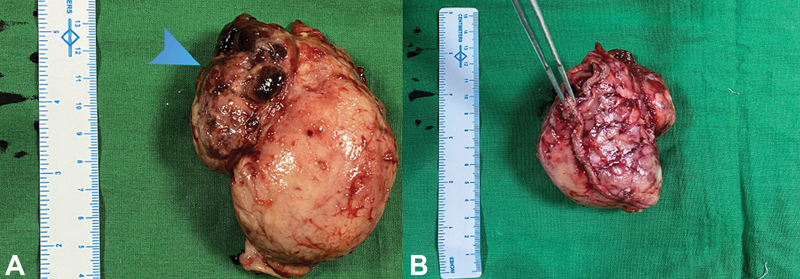
( **A** ) The resected specimen, measuring 9.5 × 8.5 × 7.5 cm, with the arrowhead pointing to the mucosal ulceration; ( **B** ) The resected specimen with the forceps holding the staple line.

**Operative steps** : Under general anesthesia, the patient was positioned supine with his legs split. Laparoscopic ports were inserted as shown in Fig. 3 . The initial diagnostic laparoscopy did not reveal any peritoneal involvement. The lesser sac was accessed by dividing the gastrocolic and posterior gastric attachments. The tumor mass was large, making gastric retraction difficult. The tumor base at the posterior gastric wall exhibited increased vascularity ( Fig. 4 ) without any evidence of invasion into adjacent tissues. After replacing the stomach in its natural position, a liberal, longitudinal anterior gastrotomy was created at the summit of the tumor using ultrasonic shears. Manipulation of the tumor mass had caused brisk bleeding from the ulcer, which could not be controlled with bipolar energy. The tumor was delivered through the gastrotomy ( Fig. 5 ) and pivoted over the shaft of a grasper placed parallel to the splenic axis. This caused the gastric wall adjacent to the tumor base to tent ( Fig. 6a ), allowing us to apply several linear staplers across it ( Fig. 6b ). All sequential stapler fires were applied in close apposition to the tumor, with the intention to save as much of the uninvolved posterior gastric wall. The use of staplers not only allowed us to resect the lesion, which halted bleeding, but also simultaneously reconstructed the posterior gastric wall. The staple line was oversewn on its luminal aspect with 2–0 polydiaxonone suture in a continuous manner after the resected lesion was placed in an endobag and parked adjacent to the right hepatic lobe. The anterior gastrotomy was closed using 2–0 delayed-absorbable barbed suture in two layers. The patient was placed in a steep Trendelenburg position, and saline was instilled into the upper abdomen. Intraoperative gastroscopy with intraluminal CO2 insufflation helped objectively confirm the integrity of the gastrotomy closures. The saline was suctioned out and a drain was placed adjacent to the stomach through the flank port. The specimen was retrieved through a Pfannenstiel incision. The resected specimen measured 9.5 × 8.5 × 7.5 cm ( Fig. 7 ). The operative time was 195 minutes, and the estimated blood loss was 175 mL (see video, supplemental digital content 1, which demonstrates the clinical course of the patient and operative steps). 

The patient commenced oral fluids on the second postoperative day and a blenderized soft diet on day 4. His abdominal drain was pulled on day 4, and he was discharged from the hospital on day 5. He suffered a superficial surgical site infection (SSI) at the extraction site, which responded well to oral antibiotics. The histopathology report confirmed a GIST with negative margins, with 10to 12 mitosis per 10 high powered fields, indicating a high potential for malignancy. The tumor stained positively for CD34 and DOG-1, was weakly positive for c-kit, and negative for S100 and desmin on immunohistochemistry. After a consultation with the medical oncologist, adjuvant imatinib therapy was commenced.

## Discussion

 Gastrointestinal stromal tumors are fairly common malignancies within the GI tract. Suspected gastric GISTs should be evaluated using endoscopy, CT, and MRI scans. Although endoscopy is useful to locate and characterize the lesion, biopsies with endoscopic forceps rarely obtain adequate tissue to confirm the diagnosis, as seen in our case. 2 Endoscopic ultrasound can confirm the origin of the tumor from the submucosal layer and allow for image-guided, deep sampling, which can confirm the cell of origin. 

**Fig. 8:**
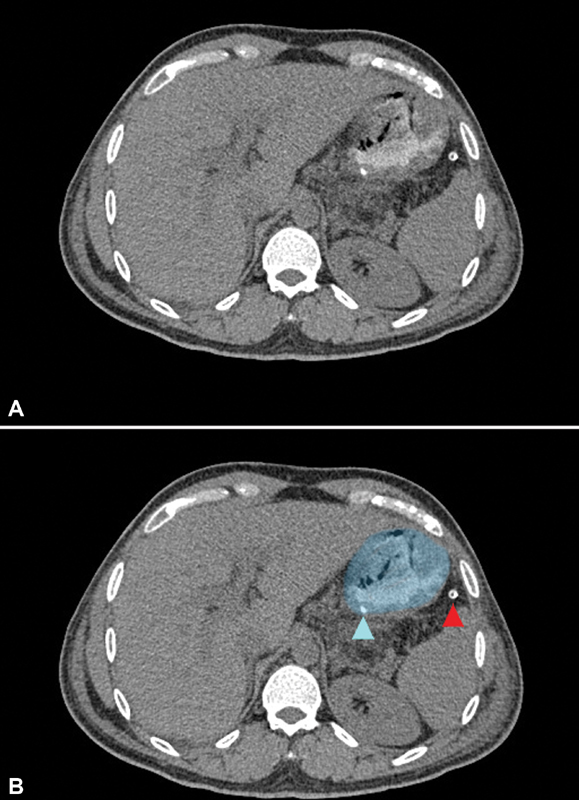
( **A** ) A postoperative computed tomography (CT) scan with luminal contrast was performed to check the integrity of gastric closure. In ( **B** ), the same scan has the stomach highlighted in blue. The posterior staple line is indicated by the blue arrowhead. There is no discernable luminal narrowing at this level, which is an advantage of the transgastric approach over a wedge gastrectomy. The red arrowhead indicates the drain placed at surgery.

 Complete surgical resection remains the mainstay of treatment for nonmetastatic GIST. It is the only potentially curative therapy. Because of favorable outcomes after curative resections, and the potential risk of tumor rupture and peritoneal seeding at surgery, open surgery has been the standard approach. 3 Because GISTs are not aggressively invasive and rarely metastasize via the lymph channels, conventional gastrectomies with wide lymph nodal clearance may be overzealous, with a higher risk of immediate and delayed complications. A wide, local resection with a 1 to 2 cm margin may be considered adequate. 4 Additionally, a stapled wedge gastrectomy would lead to a loss of significant uninvolved gastric wall with the potential for significant luminal compromise. In our case, a wedge resection would have produced an hourglass gastric configuration with a constriction toward the lesser curvature. With the use of a transgastric approach, we circumvented these potential adverse sequelae ( Fig. 8 ). One potential shortcoming of this approach is compromising the vascularity of the greater curvature, which now lies between two longitudinal gastric incisions. We confirmed adequate vascularity of the greater curvature in our patient with the use of indocyanine green (ICG) fluorescence angiography. 

 While traditionally GISTs were thought to exist on a spectrum from benign to malignant, it is now understood that all GISTs have some malignant potential. Tumor size and mitotic index are the two most significant attributes, which help stratify malignant potential of the tumor. 5 Gastric GISTs are usually less aggressive. It has also been seen that the margin of resection does not significantly affect the outcome, with similar recurrence-free survival in patients who had R0 or R1 resections. 6 Since these tumors rarely spread to the lymph nodes, a lymphadenectomy is seldom required. Keeping these features in mind, these tumors are good candidates for minimal access surgery. 7

 Up until a few years ago, laparoscopic surgery was only considered for smaller GISTs. Since then, studies have shown that as long as the aforementioned oncological principles are followed, laparoscopic surgery is not only feasible, but has better short-term outcomes in terms of decreased blood loss and shorter hospital stay. 8 Further, a recent meta-analysis showed that long-term outcomes were found to be equivalent to open surgery, even for larger GISTs. 9

## Conclusion

So, minimally invasive surgery can be considered as the first approach for uncomplicated cases, irrespective of their size. Appropriate patient selection and advanced laparoscopic skills are critical to ensure that oncologic principles in the management of GIST of the stomach are not compromised.
